# Survival outcome among patients with out-of-hospital cardiac arrest who received cardiopulmonary resuscitation in China: a systematic review and meta-analysis

**DOI:** 10.1186/s40001-022-00955-x

**Published:** 2023-01-04

**Authors:** Guozhong Zhou, Yan Wang, Zihong Sun, Mingqi Yuan, Yunlin Ma, Qianxi Wu, Chunyan Wu, Jing Xu, Yongyi Li, Yunchuan Liu, Zhenzhou Wang, Chao Song

**Affiliations:** 1 Department of Science and Research, Anning First People’s Hospital Affiliated to Kunming University of Science and Technology, Kunming, 650302 Yunnan China; 2 Department of Emergency Medicine, Anning First People’s Hospital Affiliated to Kunming University of Science and Technology, Kunming, 650302 Yunnan China; 3 School of Basic Medical Sciences, Kunming Medical University, Kunming, 650051 Yunnan China; 4 Intensive Care Unit, Anning First People’s Hospital Affiliated to Kunming University of Science and Technology, Kunming, 650302 Yunnan China; 5 Emergency Center, Anning First People’s Hospital Affiliated to Kunming University of Science and Technology, Kunming, 650302 Yunnan China; 6 Emergency Center of Yunnan Province, Kunming, 650031 Yunnan China; 7 Nursing Department, Anning First People’s Hospital Affiliated to Kunming University of Science and Technology, Kunming, 650302 Yunnan China; 8 Department of Medical Imaging, Anning First People’s Hospital Affiliated to Kunming University of Science and Technology, Kunming, 650302 Yunnan China

**Keywords:** Out-of-hospital cardiac arrest, Cardiopulmonary resuscitation, Survival outcome, Emergency medical services, Meta-analysis

## Abstract

**Background:**

This study aimed to assess the survival outcomes among patients with out-of-hospital cardiac arrest (CA) who received cardiopulmonary resuscitation (CPR) in China.

**Methods:**

Relevant studies, published between January 1, 2010 and September 5, 2022, were retrieved from databases, including EMBASE, PubMed, Cochrane Library, the China Biology Medicine disk, China National Knowledge Infrastructure, and Wanfang databases. We included clinical studies in which all patients were diagnosed with CA and underwent out-of-hospital CPR, and the outcome variables were at least one of the following: return of spontaneous circulation (ROSC), survival to admission, survival to hospital discharge, 1-month survival, achieved good neurological outcomes, and 1-year survival. Two investigators independently extracted the study data and assessed its quality using a modified Newcastle–Ottawa Scale tool. The data were pooled using random-effects models.

**Results:**

Of the 3620 identified studies, 49 (63,378 patients) were included in the meta-analysis. The pooled ROSC rate was 9.0% (95% confidence interval [CI] 7.5–10.5%, *I*^*2*^ = 97%), the pooled survival to admission rate was 5.0% (95% CI 2.7–8.0%, *I*^*2*^ = 98%), and the pooled survival to discharge rate was 1.8% (95% CI 1.2–2.5%, *I*^*2*^ = 95%). Additionally, the ROSC rate of patients with bystander CPR was significantly higher than that of those without bystander CPR, and the pooled odds ratio (OR) was 7.92 (95% CI 4.32–14.53, *I*^*2*^ = 85%). The ROSC rate of participants who started CPR within 5 min was significantly higher than that of those who started CPR after 5 min, and the pooled OR was 5.92 (95% CI 1.92–18.26, *I*^*2*^ = 85%). The ROSC rate of participants with defibrillation was significantly higher than that of those without defibrillation, and the pooled OR was 8.52 (95% CI 3.72–19.52, *I*^*2*^ = 77%).

**Conclusion:**

The survival outcomes of out-of-hospital CPR in China are far below the world average. Therefore, the policy of providing automated external defibrillators (AEDs) in public places and strengthening CPR training for healthcare providers and public personnel should be encouraged and disseminated nationwide.

*Trial registration* This study was registered in PROSPERO (CRD42022326165) on 29 April 2022.

**Supplementary Information:**

The online version contains supplementary material available at 10.1186/s40001-022-00955-x.

## Background

Out-of-hospital cardiac arrest (CA) is a major public health challenge [1]. In China, there are more than 230 million people with cardiovascular disease, and 550,000 individuals experience CA every year [2]. However, survival after out-of-hospital CA is poor. For example, in Beijing, the capital of China and where medical technology was well developed, only 1.3% of the patients with out-of-hospital CA were discharged alive, and only 1.0% had a favorable neurological outcome in 2012 [3]. The outcomes reported in other cities in China were similar [4, 5]. Therefore, the survival rate of out-of-hospital CA in China is estimated at less than 1%, which has been widely cited in many previous studies [2, 6–11]. Nevertheless, evidence from meta-analysis is still lacking.

In 2016, Gu et al. published a meta-analysis with 57 included studies and reported the pooled “heartbeat recovery rate” in patients with out-of-hospital CA was 17.1% [12]. In 2020, Gu et al. published an updated meta-analysis with 116 studies and reported similar results [13]. However, because of the improper inclusion and exclusion criteria, many low-quality studies were included in these two meta-analyses, and overestimation of the outcomes of out-of-hospital cardiopulmonary resuscitation (CPR) was inevitable. Moreover, the outcomes of “heartbeat recovery” or “success rate” used in the two studies were confusing.

Using uniform terms and definitions to assess survival outcomes for out-of-hospital resuscitation is the premise to compare the outcomes inter- and intrasystem and drive to quality improvement [14]. In 1991, the international guidelines for reporting and registering the outcomes of out-of-hospital CA were published, namely, Utstein reporting model [14], which was upgraded and simplified twice in 2004 [15] and 2015 [16]. Therefore, a meta-analysis using the international Utstein reporting model is necessary to assess the survival rate of patients with out-of-hospital CA who received CPR in China.

## Materials and methods

We performed this meta-analysis according to the preferred reporting items for systematic reviews and meta-analyses guidelines [17]. This study was registered in PROSPERO (CRD42022326165) on 29 April 2022.

### Literature retrieval

Relevant studies, published between January 1, 2010 and September 5, 2022, were retrieved from databases, including EMBASE, PubMed, Cochrane Library, the China Biology Medicine disk, China National Knowledge Infrastructure, and Wanfang databases. The English keywords were “Out-of-Hospital Cardiac Arrest,” “Cardiopulmonary Resuscitation,” and “China [Affiliation].” The Chinese keywords were “心脏骤停” and “心肺复苏.” In addition, reference lists of the relevant articles were manually checked for other potentially relevant papers.

### Inclusion and exclusion criteria

The inclusion criteria were as follows: (1) the study population was composed of adults (age ≥ 18 years); (2) patients with CA that caused by heart problem; (3) patients were diagnosed with CA and underwent out-of-hospital CPR; and (4) the outcome variables were at least one of the following: return of spontaneous circulation (ROSC), survival to admission, survival to hospital discharge, 1-month survival, achieved good neurological outcomes, and 1-year survival. The definition of CA and these outcomes is shown in Additional file 1: Table S1.

The exclusion criteria were as follows: (1)  ≥  20% of patients were children; (2)  ≥  20% of patients with specific causes of CA, such as trauma, myocardial infarction, or drowning; (3) studies in that only specific patients were selected, such as patients with mechanical compression or hands-only CPR; (4) with the absence or ambiguous definitions of CA; (5) with the absence or ambiguous definitions of survival outcomes, such as “successful recovery” or “heartbeat recovery”; and (6) abstract, reviews, case reports, case–control studies, and animal studies.

Two investigators (YW and ZHS) independently screened article titles and abstracts retrieved from the literature search. Full texts of potentially eligible studies were further assessed for final inclusion. A third investigator (GZZ) cross-checked extracted data, and disagreements were resolved through consensus.

### Data extraction and quality evaluation

For each paper, the first author, year of publication, country or region of publication, sample size, sex, age, and other patient-related data were extracted. The ROSC, survival to admission, survival to hospital discharge, 1-month survival, and achieved good neurological outcomes were also analyzed as outcome variables. Two investigators (YW and ZHS) independently extracted data from individual studies. Full texts of potentially eligible studies were further assessed for final inclusion. A third investigator (GZZ) cross-checked extracted data, and disagreements were resolved through consensus.

A modified version of Newcastle–Ottawa Scale (NOS) was used to assess the quality of each study. Studies with NOS score of 1–2, 3–4, and 5–6 were considered of low, intermediate, and high quality, respectively. Two investigators (YW and ZHS) independently assessed the methodological quality of a quarter of the studies, and the third investigator (GZZ) independently reviewed those assessments. Disagreements were resolved through consensus.

### Statistical analysis

A random-effects model was used to calculate pooled results and a 95% confidence interval (CI). *I*^*2*^ statistic was used to assess heterogeneity of included studies [18], with *I*^*2*^ > 50% suggesting significant heterogeneity. “Acsine” test was set as a parameter in publication bias detection. All *P*-values were two sided. A *P*-value < 0.05 was considered statistically significant. This meta-analysis was conducted using the “meta” package in R statistical software version 3.4.3 (Schwarzer, 2007; Team, 2017).

## Results

### Characteristics of the selected studies

**Fig. 1:**
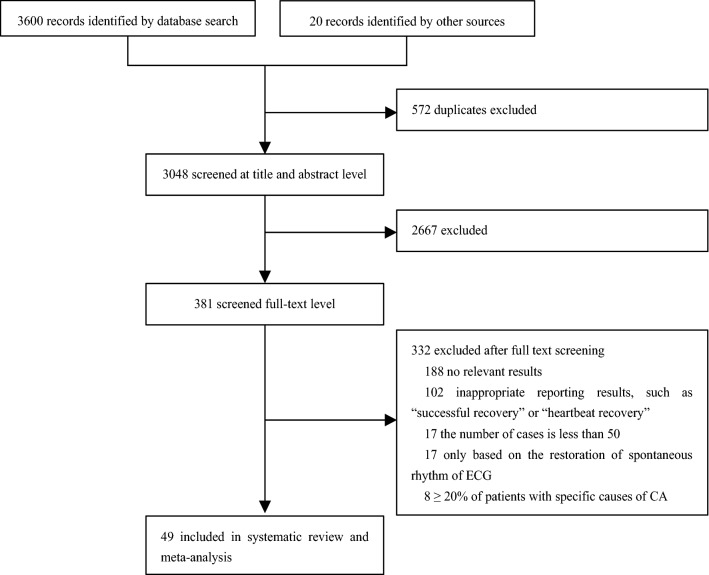
Study selection

A total of 3620 papers were retrieved. After excluding duplicate and irrelevant papers, a total of 49 studies, including 63,378 patients, were included. The literature retrieval flow chart is shown in Fig. 1.

Of the 49 studies, 42 were from Eastern China, and 7 were from Western China. Additionally, 25 studies included adults, 15 included adults and children, and 9 did not state the age of the participants. Only 20 studies reported information on bystander CPR, and only 6 stated that more than 20 participants received bystander CPR. For detailed characteristics of study individuals, see Additional file 1: Table S2.

The characteristics of the included papers are shown in Additional file 1: Table S2. The results of the evaluation of the study quality, according to the NOS scale, are shown in Additional file 1: Fig. S1.

### Return of spontaneous circulation (ROSC)

**Fig. 2:**
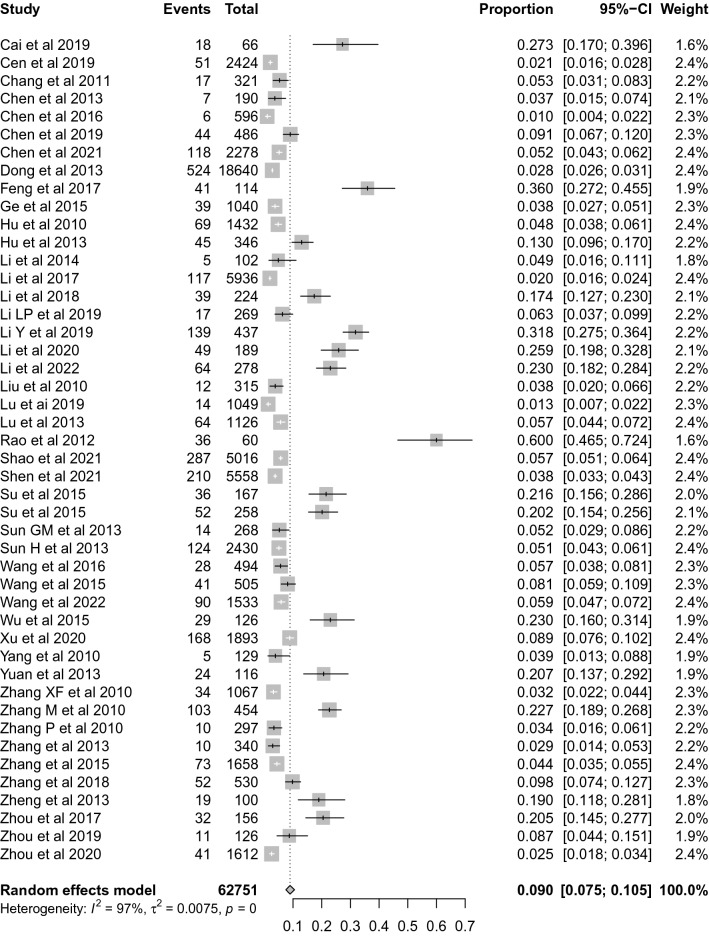
Forest plot of the ROSC rate for out-of-hospital CA patients who received CPR in China

A total of 46 studies with 62,751 participants were eligible for the calculation of the ROSC rate, and the pooled rate was 9.0% (95% CI 7.5–10.5%, *I*^*2*^ = 97%), see Fig. 2. Subgroup analyses showed that the ROSC rate of group with less than 500 participants was significant more than that of group with participants of more than 500, with pooled rate of (14.4%; 95% CI 10.5–18.7%, *I*^*2*^ = 96%) and (4.2%; 95% CI 3.4–5.1%, *I*^*2*^ = 96%), respectively. In addition, subgroup analyses also showed differences in the ROSC rate by age of participants and proportion of bystander CPR, however, the 95% CIs were overlapped, See Table 1.

**Table 1:** ROSC, survival to admission, survival to discharge, achieved good neurological outcomes, and 1-month survival: the overall estimates and subgroup analyses

	Studies (*n*)	Participants (*n*)	Rate (%)	*I*^*2*^ (%)
ROSC	46	62,751	9.0 (7.5–10.5)	97
Year of publication				
2010–2015	24	31,487	8.8 (7.5–10.5)	97
2015–2022	22	31,264	9.3 (7.0–11.8)	98
Area				
Eastern of China	39	59,619	8.5 (7.1–10.1)	97
Western of China	7	3261	10.9 (4.9–18.8)	95
Age of the participants				
Adults	24	18,821	11.4 (8.9–14.3)	97
Adults and children	14	26,950	5.9 (4.1–8.0)	96
NS	8	16,980	7.9 (5.2–11.0)	97
Proportion of males				
Less than 60%	16	14,225	10.9 (7.7–14.5)	97
More than 60%	24	19,387	8.7 (6.7–10.8)	96
NS	6	29,139	6.3 (3.6–9.8)	99
Proportion of bystander CPR				
Less than 20%	13	14,017	4.6 (3.1–6.4)	96
More than 20%	5	9192	8.7 (5.8–12.2)	95
NS	28	39,542	11.8 (9.3–14.6)	98
Participants				
Less than 500	27	6428	14.4 (10.5–18.7)	96
More than 500	19	56,323	4.2 (3.4–5.1)	96
Quality				
Middle	20	27,326	9.9 (7.8–12.4)	97
High	26	35,425	8.3 (6.2–10.6)	98
Survival to admission	10	13,922	5.0 (2.7–8.0)	98
Year of publication				
2010–2015	4	3139	3.2 (0.0–11.2)	98
2016–2022	6	10,783	6.3 (3.2–10.4)	98
Area				
Eastern of China	8	11,307	6.0 (3.2–9.7)	98
Western of China	2	2615	1.9 (0.0–9.9)	96
Age of the participants				
Adults	7	10,968	6.2 (3.3–9.9)	98
Adults and children	2	2764	4.1 (0.0–24.0)	99
NS	1	190	0.5 (0.0–2.1)	–
Percentage of males				
More than 60%	8	11,454	6.2 (3.0–10.4)	98
NS	2	2468	1.8 (0.1–5.9)	89
Bystander CPR				
Less than 20%	3	3330	3.3 (0.6–8.0)	97
More than 20%	2	7294	2.4 (0.9–4.5)	95
NS	5	3298	8.4 (0.8–23.2)	99
Participants				
Less than 500	5	1035	10.6 (3.2–21.6)	96
More than 500	5	12,869	1.8 (0.6–3.6)	97
Quality				
Middle	6	5695	5.3 (1.3–11.6)	98
High	4	8227	4.3 (1.7–7.9)	97
Survival to discharge	18	43,905	1.8 (1.2–2.5)	95
Year of publication				
2010–2015	5	19,540	2.4 (0.4–6.0)	95
2015–2022	13	24,365	1.7 (1.0–2.6)	95
Area				
Eastern of China	18	43,905	1.8 (1.2–2.5)	95
Age of the participants				
Adults	11	13,025	1.9 (1.1–2.9)	92
Adults and children	2	18,974	1.8 (0–8.7)	97
NS	5	11,906	2.0 (0.8–3.8)	95
Percentage of males				
Less than 60%	4	6,841	0.9 (0.2–2.1)	85
More than 60%	10	10,020	3.6 (2.0–5.6)	94
NS	4	27,044	0.5 (0.2–1.0)	93
Bystander CPR				
Less than 20%	3	4194	0.7 (0.1–1.9)	92
More than 20%	5	7642	2.5 (1.0–4.8)	93
NS	10	32,069	2.1 (1.2–3.2)	95
Participants				
Less than 500	9	1753	4.3 (2.0–7.4)	87
More than 500	9	42,152	0.7 (0.4–1.2)	94
Quality				
Middle	8	22,371	3.4 (1.5–6.2)	97
High	10	21,534	1.0 (0.5–1.6)	92
Achieved good neurological outcomes	3	2267	2.5 (0.2–7.0)	94
One-month survival	2	1154	2.7 (0.0–14.5)	95

*ROSC* return of spontaneous circulation, *NS* not specified, *CPR* cardiopulmonary resuscitation

### Survival to admission

**Fig. 3:**
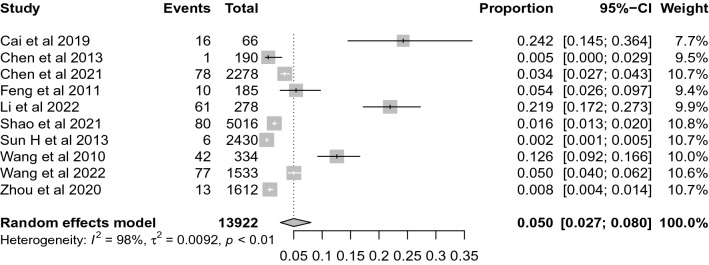
Forest plot of the survival to admission rate for out-of-hospital CA patients who received CPR in China

Ten studies with 13,922 participants were eligible for the comparison of the calculation of survival to admission rate, and the pooled rate was 5.0% (95% CI 2.7–8.0%, *I*^*2*^ = 98%), see Fig. 3. Subgroup analyses showed differences in survival to admission rate by year of publication, area, proportion of bystander CPR, and number of participants, however, the 95% CIs were overlapped, see Table 1.

### Survival to discharge

**Fig. 4:**
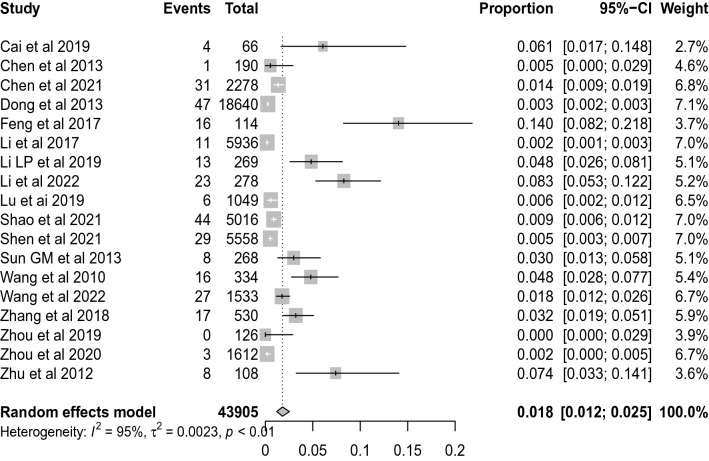
Forest plot of the survival to discharge rate for out-of-hospital CA patients who received CPR in China

Eighteen studies with 43,905 participants were eligible for the comparison of calculation of the survival to discharge rate, and the pooled rate was 1.8% (95% CI 1.2–2.5%, *I*^*2*^ = 95%), see Fig. 4. Subgroup analyses showed that the survival to discharge rate of group with less than 500 participants was significant more than that of group with participants of more than 500, with pooled rate of (4.3%; 95% CI 2.0–7.4%, *I*^*2*^ = 87%) and (0.7%; 95% CI 0.4–1.2%, *I*^*2*^ = 94%), respectively. In addition, Subgroup analyses also showed differences in survival to discharge rate by the year of publication, proportion of males, proportion of bystander CPR and quality of the included studies, however, the 95% CIs were overlapped, see Table 1.

### One-month survival and achieved good neurological outcomes

Only two studies with 1,154 participants were eligible for the comparison of calculation of the 1-month survival rate, and the pooled rate was 2.7% (95% CI 0.0–14.5%, *I*^*2*^ = 95%). Likewise, only three studies with 2267 participants were eligible for the comparison of calculation of achieved good neurological outcomes rate, and the pooled rate was 2.5% (95% CI 0.2–7.0%, *I*^*2*^ = 94%), see Table 1.

### Contributors for ROSC rate

As shown in Table 2 and Additional file 1: Figs. S1–S4, the ROSC rate of participants with bystander CPR was significantly higher than that of those without bystander CPR, and the pooled OR was 7.92 (95% CI 4.32–14.53, *I*^*2*^ = 85%). The ROSC rate of the participants who started CPR within 5 min was significantly higher than that of those who started CPR after 5 min, and the pooled OR was 5.92 (95% CI 1.92–18.26, *I*^*2*^ = 85%). The ROSC rate of participants with defibrillation was significantly higher than that of those without defibrillation, and the pooled OR was 8.52 (95% CI 3.72–19.52, *I*^*2*^ = 77%). However, there was no difference between participants with and without advanced airways, and the pooled OR was 1.12 (95% CI 0.84–1.78, *I*^*2*^ = 76%).

**Table 2:** Influence of bystander CPR, start CPR time, defibrillation, and advanced airway on ROSC

	Studies (*n*)	Participants (*n*)	Rate (%)	*I*^*2*^ (%)	OR	*I*^*2*^ (%)
Bystander CPR						
Bystander CPR	6	2154	23.5 (13.4–35.4)	91	7.92 (4.32–14.53)	85
No Bystander CPR	6	21,312	4.2 (1.7–7.8)	98		
Start CPR time						
Less than 5 min	6	621	16.4 (2.5–39.2)	97	5.92 (1.92–18.26)	85
More than 5 min	6	2114	2.9 (1.0–5.6)	89		
Less than 10 min	6	2791	10.8 (4.9–18.5)	96	2.88 (1.12–7.43)	80
More than 10 min	6	133	3.0 (0.7–6.8)	86		
Defibrillation						
Defibrillation	4	209	32.9 (16.5–51.8)	86	8.52 (3.72–19.52)	77
No defibrillation	4	4940	6.0 (2.0–11.9)	98		
Advanced airway						
Advanced airway	13	5975	10.6 (7.4–14.2)	92	1.22 (0.84–1.78)	76
No advanced airway	13	8107	9.4 (5.8–13.7)	97		

*ROSC* return of spontaneous circulation, *OR* odds ratio, *CPR* cardiopulmonary resuscitation

### Publication bias

The funnel plot was symmetrical for the meta-analysis of the incidence of ROSC, survival to admission, and survival to discharge in patients with out-of-hospital CA who received CPR (see Additional file 1: Fig. S5). The “Acsine” test revealed no evidence of publication bias in survival to admission (*P* = 0.1284) and survival to discharge (*P* = 0.0883). However, there was a publication bias in ROSC (*P* = 0.0011).

## Discussion

To the best of our knowledge, this is the first meta-analysis to assess survival outcomes among out-of-hospital CPR using Utstein reporting model in China. We found that the pooled ROSC rate was 9.0% (95% CI 7.5–10.5%, *I*^*2*^ = 97%), the pooled survival to admission rate was 5.0% (95% CI: 2.7–8.0%, *I*^*2*^ = 98%), and the pooled survival to discharge rate was 1.8% (95% CI 1.2–2.5%, *I*^*2*^ = 95%).

In 2020, Yan et al. assessed the global survival rate among adult out-of-hospital CPR and found that the pooled ROSC rate was 29.7% (95% CI 27.6–31.7%), the pooled survival to admission rate was 22.0% (95% CI 20.7–23.4%), and the pooled survival to discharge rate was 8.8% (95% CI 8.2–9.4%)[19]. Even in Asia, which has the lowest survival rate, the pooled ROSC rate was 22.1% (95% CI 18.1–26.0%), the pooled survival to admission rate was 15.6% (95% CI 13.2–18.0%), and the pooled survival to discharge rate was 4.5% (95% CI 3.1–5.9%)[19]. Therefore, our study found very frustrating results; that is, the survival outcomes of out-of-hospital CA in China are far below the world average.

It should be pointed out that almost all the included studies were from large or medium cities, irrespective of the eastern or western regions. Currently, few studies from small cities or rural areas in China have been published. Moreover, our subgroup analysis found that survival rates were affected by the number of participants in our meta-analysis, and the group with participants more than 500 was lower than that of group with participants less than 500. Large sample studies usually have better research designs and provide more reliable results [4, 20, 21]. Therefore, the actual survival outcomes in China may be lower than that of our pooled results. More pessimistically, our subgroup analysis showed no difference in the survival outcomes between the 2010–2015 and 2016–2022 groups, which means that the survival rate of out-of-hospital CA might not have increased in the past 12 years.

Our meta-analysis revealed that the ROSC rate of those who started CPR within 5 min was 5.92 times that of those who started CPR after 5 min. In addition, the ROSC rate of those with bystander CPR was 7.92 times that of those without bystander CPR. Our results support that early bystander CPR is a key determinant of survival [22–25]. However, the implementation rate for bystander CPR in China is low (11.4% in Beijing [3, 26], 8.83% in Zhenzhou [27], and 0.59% in Hefei [4], *vs.* 39.4% in the United States [28] and 39% in Australia [29]) since the prevalent training rate in China is less than 1% and skill retention training is also rare [2]. Our meta-analysis also revealed that the ROSC rate of those with defibrillation was 8.52 times that of those without defibrillation. Recently, the Chinese government has begun to attach importance to out-of-hospital CA. Provision of automated external defibrillators in public places and financial support for public training provide the possibility to improve the survival rate in the future.

In Gu et al.’s meta-analysis [12, 13], unclear outcomes of “heartbeat recovery” or “success rate” were used, which reflected that researchers were unfamiliar with Utstein reporting model. In fact, we conducted a survey on the perception of Utstein model among Chinese healthcare providers in 2017 [30]; 41.2% of 10,224 participants reported that they had not heard of Utstein model. In addition, Chinese healthcare providers always use “successful resuscitation” to assess the outcome of CPR. However, the understanding of the term is confused in China. In the survey, 40.9%, 23.1%, and 21.6% of Chinese medical staff considered the recovery of spontaneous rhythm, pulse, and breathing as “successful resuscitation,” respectively. Therefore, it is urgent to strengthen the perception training and application of Utstein registration model in China.

## Limitation

The meta-analysis had several limitations. First, most included studies were from large or medium cities. Second, most included studies were retrospective observational studies. Third, while obvious heterogeneity was present in several groups, subgroup analyses were not possible to identify the source of heterogeneity.

## Conclusion

The survival outcomes of out-of-hospital CA in China are far below the world average. Therefore, providing AED in public places and strengthening CPR training for healthcare providers and public personnel should be encouraged and disseminated nationwide.

## Supplementary Information

**Additional file 1: Table S1.** Definition of survival outcomes. **Table S2.** Detailed individual study characteristics. **Figure S1.** The quality of included studies. **Figure S2.** Forest plot of the odds ratio of ROSC rate with bystander CPR vs. without bystander CPR. **Figure S3.** Forest plot of the odds ratio of ROSC rate in start CPR time within 5 minutes vs. more than 5 minutes. **Figure S4.** Forest plot of the odds ratio of ROSC rate with defibrillation vs. without defibrillation. **Figure S5.** Forest plot of the odds ratio of ROSC rate with advanced airway (AA) vs. without advanced airway. Figure S6 Funnel plot of ROSC rate, survival to admission rate and survival to discharge rate ROSC rate (A), survival to admission rate (B), and survival to discharge rate (C).

## Data Availability

Data may be made available by contacting the corresponding author.

## Abbreviations

AED: Automated external defibrillator
CA: Cardiac arrests
CPR: Cardiopulmonary resuscitation
CI: Confidence interval
NOS: NEWCASTLE–Ottawa Scale tool
OR: Odds ratio
ROSC: Return of spontaneous circulation
