# Supplementary material for: Survival outcome among patients with out-of-hospital cardiac arrest who received cardiopulmonary resuscitation in China: a systematic review and meta-analysis
Source: Eur J Med Res. 2023 Jan 4;28:8. doi: 10.1186/s40001-022-00955-x (PMC9811716; doi:10.1186/s40001-022-00955-x)
Supplement: Supplementary file 1 — Additional file 1: Table S1. Definition of survival outcomes. Table S2. Detailed individual study characteristics. Figure S1. The quality of included studies. Figure S2. Forest plot of the odds ratio of ROSC rate with bystander CPR vs. without bystander CPR. Figure S3. Forest plot of the odds ratio of ROSC rate in start CPR time within 5 minutes vs. more than 5 minutes. Figure S4. Forest plot of the odds ratio of ROSC rate with defibrillation vs. without defibrillation. Figure S5. Forest plot of the odds ratio of ROSC rate with advanced airway (AA) vs. without advanced airway. Figure S6 Funnel plot of ROSC rate, survival to admission rate and survival to discharge rate ROSC rate (A), survival to admission rate (B), and survival to discharge rate (C). [file 40001_2022_955_MOESM1_ESM.doc]

**Supplementary web appendix for:**

**Survival outcome among out-of-hospital cardiac arrest patients who received cardiopulmonary resuscitation in China: a meta-analysis and systematic review**

[Figure S1 The quality of included studies 2](#__RefHeading___Toc13317)

[Figure S2 Forest plot of the odds ratio of ROSC rate with bystander CPR *vs.* without bystander CPR 3](#__RefHeading___Toc18601)

[Figure S3 Forest plot of the odds ratio of ROSC rate in start CPR time within 5 minutes *vs.* more than 5 minutes 3](#__RefHeading___Toc17651)

[Figure S4 Forest plot of the odds ratio of ROSC rate with defibrillation *vs.* without defibrillation 3](#__RefHeading___Toc4969)

[Figure S5 Forest plot of the odds ratio of ROSC rate with advanced airway (AA) *vs.* without advanced airway 4](#__RefHeading___Toc18489)

[Figure S6 Funnel plot of ROSC rate, survival to admission rate and survival to discharge rate 4](#__RefHeading___Toc19107)

[Table S1 Definition of survival outcomes 6](#__RefHeading___Toc7299)

[Table S2 Detailed individual study characteristics 7](#__RefHeading___Toc10361)

[References 13](#__RefHeading___Toc6408)




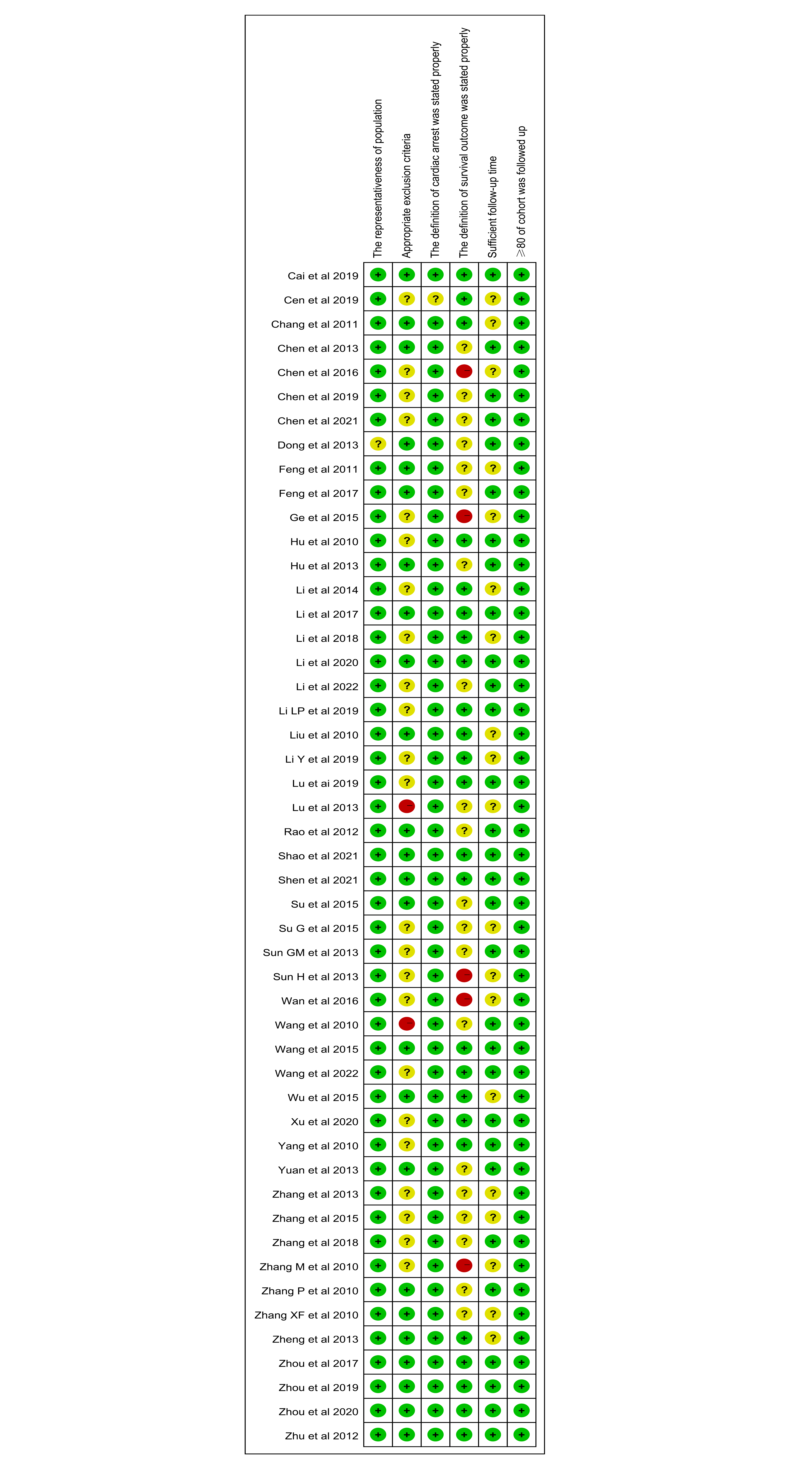


[Figure S1.](#__RefHeading___Toc31087) The quality of included studies.





Figure S2. Forest plot of the odds ratio of ROSC rate with bystander CPR *vs.* without bystander CPR.





Figure S3.. Forest plot of the odds ratio of ROSC rate in start CPR time within 5 minutes *vs.* more than 5 minutes.










Figure S4. Forest plot of the odds ratio of ROSC rate with defibrillation *vs.* without defibrillation.











Figure S5. Forest plot of the odds ratio of ROSC rate with advanced airway (AA) *vs.* without advanced airway.

A B







C





Figure S6. Funnel plot of ROSC rate, survival to admission rate and survival to discharge rate.

ROSC rate (A), survival to admission rate (B) and survival to discharge rate (C)

Table S1. Definition of survival outcomes.

| Terms | | Definition |
| --- | --- | --- |
| Cardiac Arrest | |  |
| Initial survival | ROSC | A brief (approximately >30 seconds) restoration of spontaneous circulation that provides evidence of more than an occasional gasp, occasional fleeting palpable pulse, or arterial waveform. |
|  | Survival to admission | ROSC sustained until arrival at the emergency department and transfer of care to medical staff at the receiving hospital |
| Long-term survival | Survival to discharge | Survival to hospital discharge is the point at which the patient is discharged from the hospital’s acute care unit regardless of neurological status, outcome, or destination. |
|  | 1-month survival | Survival to 30 days. |
|  | Achieved good neurological outcomes | Cerebral performance category score of 1 or 2. |
|  | 1-year survival | Survival to one year. |

Note: ROSC, return of spontaneous circulation.

Table S2. Detailed individual study characteristics.

|  | Area | Age | Male (%) | B-CPR (%) | Subgroup | Participants (n) | ROSC (n) | Survival to admission (n) | Survival to discharge (n) | One-month survival (n) | Good neurological outcomes (n) | NOS  Score |
| --- | --- | --- | --- | --- | --- | --- | --- | --- | --- | --- | --- | --- |
| Cai et al 2019[1] | Zhongshan, Guangdong | Adults | 78.79 | NS | All | 66 | 18 | 16 | 4 | - | - | 5 |
| Cen et al 2019[2] | Zhengzhou, Henan | NS | 56.93 | 8.83 | All | 2424 | 51 | - | - | - | - | 5 |
| Cen et al 2019[2] | Zhengzhou, Henan | NS | 56.93 | 8.83 | Start CPR ≦ 10 min | 1628 | 37 | - | - | - | - | 5 |
| Cen et al 2019[2] | Zhengzhou, Henan | NS | 56.93 | 8.83 | Start CPR > 10 min | 796 | 14 | - | - | - | - | 5 |
| Cen et al 2019[2] | Zhengzhou, Henan | NS | 56.93 | 8.83 | Defibrillation | 84 | 14 | - | - | - | - | 5 |
| Cen et al 2019[2] | Zhengzhou Henan | NS | 56.93 | 8.83 | No defibrillation | 2340 | 37 | - | - | - | - | 5 |
| Cen et al 2019[2] | Zhengzhou, Henan | NS | 56.93 | 8.83 | Balloon mask | 1776 | 23 | - | - | - | - | 5 |
| Cen et al 2019[2] | Zhengzhou, Henan | NS | 56.93 | 8.83 | Trachea cannula | 594 | 14 | - | - | - | - | 5 |
| Cen et al 2019[2] | Zhengzhou, Henan | NS | 56.93 | 8.83 | Laryngeal mask | 54 | 4 | - | - | - | - | 5 |
| Chang et al 2011[3] | Qingdao, Shandong | Adults and children | 74.69 | NS | All | 321 | 17 | - | - | - | - | 4 |
| Chang et al 2011[3] | Qingdao, Shandong | Adults and children | 74.69 | NS | Start CPR ≦ 4min | 9 | 8 | - | - | - | - | 4 |
| Chang et al 2011[3] | Qingdao, Shandong | Adults and children | 74.69 | NS | Start CPR 5-6 min | 82 | 6 | - | - | - | - | 4 |
| Chang et al 2011[3] | Qingdao, Shandong | Adults and children | 74.69 | NS | Start CPR 7-8 min | 109 | 2 | - | - | - | - | 4 |
| Chang et al 2011[3] | Qingdao, Shandong | Adults and children | 74.69 | NS | Start CPR 9-10 min | 77 | 1 | - | - | - | - | 4 |
| Chang et al 2011[3] | Qingdao, Shandong | Adults and children | 74.69 | NS | Start CPR > 10 min | 44 | 0 | - | - | - | - | 4 |
| Chen et al 2013[4] | Foshan, Guangdong | NS | NS | NS | All | 190 | 7 | 1 | 1 |  | - | 5 |
| Chen et al 2016[5] | Zhenjiang, Jiangsu | Adults and children | 68.79 | 5.7 | All | 596 | 6 | - | - | - | - | 3 |
| Chen et al 2019[6] | Shanghai | Adults | 54.53 | 14.81 | All | 486 | 44 | - | - | - | - | 4 |
| Chen et al 2019[6] | Shanghai | Adults | 54.53 | 14.81 | Defibrillation | 59 | 12 | - | - | - | - | 4 |
| Chen et al 2019[6] | Shanghai | Adults | 54.53 | 14.81 | No defibrillation | 427 | 32 | - | - | - | - | 4 |
| Chen et al 2019[6] | Shanghai | Adults | 54.53 | 14.81 | Bystander CPR | 72 | 16 | - | - | - | - | 4 |
| Chen et al 2019[6] | Shanghai | Adults | 54.53 | 14.81 | No bystander CPR | 414 | 28 | - | - | - | - | 4 |
| Chen et al 2021[7] | Beijing | Adults | NS | 21.25 | All | 2278 | 118 | 78 | 31 | - | - | 6 |
| Dong et al 2013[8] | Shanghai | Adults and children | NS | NS | All | 18640 | 524 | - | 47 | - | - | 4 |

Continue

|  | Area | Age | Male (%) | B-CPR (%) | Subgroup | Participants (n) | ROSC (n) | Survival to admission (n) | Survival to discharge (n) | One-month survival (n) | Good neurological outcomes (n) | NOS  Score |
| --- | --- | --- | --- | --- | --- | --- | --- | --- | --- | --- | --- | --- |
| Dong et al 2013[8] | Shanghai | Adults and children | NS | NS | Bystander CPR | 1870 | 319 | - | - | - | - | 4 |
| Dong et al 2013[8] | Shanghai | Adults and children | NS | NS | No bystander CPR | 16770 | 205 | - | - | - | - | 4 |
| Feng et al 2011[9] | Liuzhou, Guangxi | Adults | 66.49 | 4.32 | All | 185 | - | 10 | - | - | - | 6 |
| Feng et al 2017[10] | Quzhou, Zhejiang | NS | 64.04 | 41.23 | All | 114 | 41 | - | 16 | - | 9 | 5 |
| Ge et al 2015[11] | Shanghai | NS | 53.56 | 5.87 | All | 1040 | 39 | - | - | - | 3 | 5 |
| Ge et al 2015[11] | Shanghai | NS | 53.56 | 5.87 | Bystander CPR | 61 | 9 | - | - | - | 1 | 5 |
| Ge et al 2015[11] | Shanghai | NS | 53.56 | 5.87 | No bystander CPR | 979 | 30 | - | - | - | 2 | 5 |
| Hu et al 2010[12] | Hefei, Anhui | Adults and children | 55.59 | NS | All | 1432 | 69 | - | - | - | - | 3 |
| Hu et al 2010[12] | Hefei, Anhui | Adults and children | 55.59 | NS | Trachea cannula | 676 | 19 | - | - | - | - | 3 |
| Hu et al 2010[12] | Hefei, Anhui | Adults and children | 55.59 | NS | Balloon mask | 756 | 50 | - | - | - | - | 3 |
| Hu et al 2013[13] | Hefei, Anhui | Adults and children | 65.03 | 14.74 | All | 346 | 45 | - | - | - | - | 3 |
| Hu et al 2013[13] | Hefei, Anhui | Adults and children | 65.03 | 14.74 | Start CPR < 10 min | 219 | 33 | - | - | - | - | 3 |
| Hu et al 2013[13] | Hefei, Anhui | Adults and children | 65.03 | 14.74 | Start CPR > 10 min | 127 | 12 | - | - | - | - | 3 |
| Hu et al 2013[13] | Hefei, Anhui | Adults and children | 65.03 | 14.74 | Bystander CPR | 51 | 17 | - | - | - | - | 3 |
| Hu et al 2013[13] | Hefei, Anhui | Adults and children | 65.03 | 14.74 | No bystander CPR | 295 | 28 | - | - | - | - | 3 |
| Hu et al 2013[13] | Hefei, Anhui | Adults and children | 65.03 | 14.74 | Defibrillation | 54 | 20 | - | - | - | - | 3 |
| Hu et al 2013[13] | Hefei, Anhui | Adults and children | 65.03 | 14.74 | No defibrillation | 292 | 25 | - | - | - | - | 3 |
| Hu et al 2013[13] | Hefei, Anhui | Adults and children | 65.03 | 14.74 | Trachea cannula | 221 | 30 | - | - | - | - | 4 |
| Hu et al 2013[13] | Hefei, Anhui | Adults and children | 65.03 | 14.74 | Balloon mask | 125 | 15 | - | - | - | - | 3 |
| Li et al 2014[14] | Xuanhan, Sichuan | Adults and children | 66.67 | NS | All | 102 | 5 | - | - | - | - | 5 |
| Li et al 2014[14] | Xuanhan Sichuan | Adults and children | 66.67 | NS | Start CPR ≤ 5 min | 22 | 4 | - | - | - | - | 5 |
| Li et al 2014[14] | Xuanhan Sichuan | Adults and children | 66.67 | NS | Start CPR > 5 min | 80 | 1 | - | - | - | - | 5 |
| Li et al 2017[15] | Shanghai | NS | NS | NS | All | 5936 | 117 | - | 11 | - | - | 5 |
| Li et al 2018[16] | Dongguan, Guangdong | Adults | 62.50 | NS | All | 224 | 39 | - | - | - | - | 4 |

Continue

|  | Area | Age | Male (%) | B-CPR (%) | Subgroup | Participants (n) | ROSC (n) | Survival to admission (n) | Survival to discharge (n) | One-month survival (n) | Good neurological outcomes (n) | NOS  Score |
| --- | --- | --- | --- | --- | --- | --- | --- | --- | --- | --- | --- | --- |
| Li et al 2018[16] | Dongguan, Guangdong | Adults | 62.5 | NS | Start CPR ≤ 10 min | 39 | 23 | - | - | - | - | 4 |
| Li et al 2018[16] | Dongguan, Guangdong | Adults | 62.5 | NS | Start CPR 10-20 min | 51 | 14 | - | - | - | - | 4 |
| Li et al 2018[16] | Dongguan, Guangdong | Adults | 62.5 | NS | Start CPR > 20 min | 134 | 2 | - | - | - | - | 4 |
| Li et al 2018[16] | Dongguan, Guangdong | Adults | 62.5 | NS | Throat cannula | 135 | 17 | - | - | - | - | 4 |
| Li et al 2018[16] | Dongguan, Guangdong | Adults | 62.5 | NS | No trachea cannula | 89 | 22 | - | - | - | - | 4 |
| Li LP et al 2019[17] | Haikou, Hainan | Adults | 65.8 | NS | All | 269 | 17 | - | 13 | 10 | - | 5 |
| Li LP et al 2019[17] | Haikou, Hainan | Adults | 67.13 | NS | T-CPR | 143 | 13 | - | 11 | 9 | - | 5 |
| Li LP et al 2019[17] | Haikou, Hainan | Adults | 64.29 | NS | No T-CPR | 126 | 4 | - | 2 | 1 | - | 5 |
| Li Y et al 2019[18] | Harbin, Heilongjiang | Adults | NS | NS | All | 437 | 139 | - | - | - | - | 4 |
| Li et al 2020[19] | Chengdu, Sichuan | Adults | 65.61 | NS | All | 189 | 49 | - | - | - | - | 3 |
| Li et al 2022[20] | Hefei, Anhui | Adults | 72.3 | NS | All | 278 | 64 | 61 | 23 | 11 | - | 6 |
| Liu et al 2010[21] | Tangshan, Hebei | Adults and children | 66.35 | NS | All | 315 | 12 | - | - | - | - | 4 |
| Liu et al 2010[21] | Tangshan, Hebei | Adults and children | 66.35 | NS | Start CPR 3-5 min | 77 | 7 | - | - | - | - | 4 |
| Liu et al 2010[21] | Tangshan, Hebei | Adults and children | 66.35 | NS | Start CPR 6-10 min | 203 | 5 | - | - | - | - | 4 |
| Liu et al 2010[21] | Tangshan, Hebei | Adults and children | 66.35 | NS | Start CPR 11-20 min | 26 | 0 | - | - | - | - | 4 |
| Liu et al 2010[21] | Tangshan, Hebei | Adults and children | 66.35 | NS | Start CPR > 20 min | 9 | 0 | - | - | - | - | 4 |
| Liu et al 2010[21] | Tangshan, Hebei | Adults and children | 66.35 | NS | Trachea cannula | 215 | 12 | - | - | - | - | 4 |
| Liu et al 2010[21] | Tangshan, Hebei | Adults and children | 66.35 | NS | No trachea cannula | 100 | 0 | - | - | - | - | 4 |
| Lu et ai 2019[22] | Zheng zhou, Henan | Adults | 27.64 | 4.2 | All | 1049 | 14 | - | 6 | - | - | 6 |
| Lu et ai 2019[22] | Zheng zhou, Henan | Adults | 27.64 | 4.2 | Bystander CPR | 44 | 3 | - | - | - | - | 6 |
| Lu et ai 2019[22] | Zheng zhou, Henan | Adults | 27.64 | 4.2 | No bystander CPR | 1005 | 11 | - | - | - | - | 6 |
| Lu et ai 2019[22] | Zheng zhou, Henan | Adults | 27.64 | 4.2 | Start CPR ≤ 6 min | 230 | 5 | - | - | - | - | 6 |
| Lu et ai 2019[22] | Zheng zhou, Henan | Adults | 27.64 | 4.2 | Start CPR > 6 min | 819 | 9 | - | - | - | - | 6 |
| Lu et al 2013[23] | Zhuhai, Guangdong | Adults | 62.61 | 0.71 | All | 1126 | 64 | - | - | - | - | 3 |

Continue

|  | Area | Age | Male (%) | B-CPR (%) | Subgroup | Participants (n) | ROSC (n) | Survival to admission (n) | Survival to discharge (n) | One-month survival (n) | Good neurological outcomes (n) |
| --- | --- | --- | --- | --- | --- | --- | --- | --- | --- | --- | --- |
| Rao et al 2012[24] | Shiyan, Hubei | NS | 17.18 | NS | All | 60 | 36 | - | - | - | - |
| Shao et al 2021[25] | Beijing | Adults | 72.05 | 11.4 | All | 5016 | 287 | 80 | 44 | - | - |
| Shen et al 2021[26] | Shanghai | NS | 55.04 | NS | All | 5558 | 210 | - | 29 | - | - |
| Shen et al 2021[26] | Shanghai | NS | 55.04 | NS | Balloon mask | 2597 | 72 | - | 22 | - | - |
| Shen et al 2021[26] | Shanghai | NS | 55.04 | NS | Trachea cannula | 2961 | 138 | - | 7 | - | - |
| Su et al 2015[27] | Zhanjiang, Guangdong | Adults | 53.29 | NS | All | 167 | 36 | - | - | - | - |
| Su et al 2015[27] | Zhanjiang, Guangdong | Adults | 53.29 | NS | Balloon mask | 55 | 10 | - | - | - | - |
| Su et al 2015[27] | Zhanjiang, Guangdong | Adults | 53.29 | NS | Trachea cannula | 112 | 26 | - | - | - | - |
| Su G et al 2015[28] | Zhanjiang, Guangdong | Adults | 62.02 | NS | All | 258 | 52 | - | - | - | - |
| Su G et al 2015[28] | Zhanjiang, Guangdong | Adults | 62.02 | NS | Trachea cannula | 116 | 28 | - | - | - | - |
| Su G et al 2015[28] | Zhanjiang, Guangdong | Adults | 62.02 | NS | Mouth to mouth | 76 | 15 | - | - | - | - |
| Su G et al 2015[28] | Zhanjiang, Guangdong | Adults | 62.02 | NS | Mask oxygen supply | 62 | 12 | - | - | - | - |
| Su G et al 2015[28] | Zhanjiang, Guangdong | Adults | 62.02 | NS | Other | 4 | 1 | - | - | - | - |
| Sun GM et al 2013[29] | Zhengzhou, Henan | Adults | 74.25 | NS | All | 268 | 14 | - | 8 | - | - |
| Sun H et al 2013[30] | Kunming, Yunnan | Adults and children | 70.41 | NS | All | 2430 | 124 | 6 | - | - | - |
| Wang et al 2016[31] | Hefei, Anhui | Adults and children | 70.24 | NS | All | 494 | 28 | - | - | - | - |
| Wang et al 2016[31] | Hefei, Anhui | Adults and children | 70.24 | NS | Start CPR 1-5 min | 88 | 6 | - | - | - | - |
| Wang et al 2016[31] | Hefei, Anhui | Adults and children | 70.24 | NS | Start CPR 6-10 min | 260 | 16 | - | - | - | - |
| Wang et al 2016[31] | Hefei, Anhui | Adults and children | 70.24 | NS | Start CPR 11-15 min | 102 | 6 | - | - | - | - |
| Wang et al 2016[31] | Hefei, Anhui | Adults and children | 70.24 | NS | Start CPPR > 15min | 44 | 0 | - | - | - | - |
| Wang et al 2010[32] | Jintan, Jiangsu | Adults and children | 61.08 | NS | All | 334 |  | 42 | 16 | - | - |
| Wang et al 2015[33] | Beijing | Adults | 59.8 | 17.62 | All | 505 | 41 | - | - | - | - |
| Wang et al 2022[34] | Hefei, Anhui | Adults | 67.19 | 0.59 | All | 1533 | 90 | 77 | 27 | - | - |

Continue

|  | Area | Age | Male (%) | B-CPR (%) | Subgroup | Participants (n) | ROSC (n) | Survival to admission (n) | Survival to discharge (n) | One-month survival (n) | Good neurological outcomes (n) | NOS  Score |
| --- | --- | --- | --- | --- | --- | --- | --- | --- | --- | --- | --- | --- |
| Wu et al 2015[35] | Zigong, Sichuan | Adults | 59.52 | NS | All | 126 | 29 | - | - | - | - | 3 |
| Wu et al 2015[35] | Zigong, Sichuan | Adults | 61.9 | NS | Mouth-to-mouth | 42 | 4 | - | - | - | - | 3 |
| Wu et al 2015[35] | Zigong, Sichuan | Adults | 57.14 | NS | Trachea cannula | 42 | 10 | - | - | - | - | 3 |
| Wu et al 2015[35] | Zigong, Sichuan | Adults | 59.52 | NS | Balloon mask | 42 | 15 | - | - | - | - | 3 |
| Xu et al 2020[36] | Shenzhen, Guangdong | Adults | 73.16 | 2.96 | All | 1893 | 168 | - | - | - | - | 5 |
| Xu et al 2020[36] | Shenzhen, Guangdong | Adults | 73.16 | 2.96 | Bystander CPR | 44 | 23 | - | - | - | - | 5 |
| Xu et al 2020[36] | Shenzhen, Guangdong | Adults | 73.16 | 2.96 | Bystander CPR + AED | 12 | 9 | - | - | - | - | 5 |
| Xu et al 2020[36] | Shenzhen, Guangdong | Adults | 73.16 | 2.96 | No bystander CPR | 1849 | 145 | - | - | - | - | 5 |
| Xu et al 2020[36] | Shenzhen, Guangdong | Adults | 73.16 | 2.96 | Trachea cannula | 208 | 22 | - | - | - | - | 5 |
| Xu et al 2020[36] | Shenzhen, Guangdong | Adults | 73.16 | 2.96 | No trachea cannula | 1685 | 146 | - | - | - | - | 5 |
| Yang et al 2010[37] | Hohhot, Inner Mongolia | Adults | 50.54 | NS | All | 129 | 5 | - | - | - | - | 5 |
| Yang et al 2010[37] | Hohhot, Inner Mongolia | Adults | 74.42 | NS | All | 129 | 5 | - | - | - | - | 5 |
| Yuan et al 2013[38] | Xingtai, Hebei | Adults and children | 56.03 | NS | All | 116 | 24 | - | - | - | - | 3 |
| Yuan et al 2013[38] | Xingtai, Hebei | Adults and children | 56.03 | NS | Mouth-to-mouth | 38 | 3 | - | - | - | - | 3 |
| Yuan et al 2013[38] | Xingtai, Hebei | Adults and children | 56.03 | NS | Trachea cannula | 39 | 8 | - | - | - | - | 3 |
| Yuan et al 2013[38] | Xingtai, Hebei | Adults and children | 56.03 | NS | Balloon mask | 39 | 13 | - | - | - | - | 3 |
| Zhang XF et al 2010[39] | Wuxi, Jiangsu | Adults and children | 71.53 | 4.91 | All | 1067 | 34 | - | - | - | - | 5 |
| Zhang XF et al 2010[39] | Wuxi, Jiangsu | Adults and children | 71.53 | 4.91 | Trachea cannula | 550 | 30 | - | - | - | - | 5 |
| Zhang XF et al 2010[39] | Wuxi, Jiangsu | Adults and children | 71.53 | 4.91 | No tracheal cannula | 517 | 4 | - | - | - | - | 5 |
| Zhang M et al 2010[40] | Nantong, Jiangsu | Adults and children | 59.25 | NS | All | 454 | 103 | - | - | - | - | 3 |
| Zhang M et al 2010[40] | Nantong, Jiangsu | Adults and children | 59.25 | NS | Start CPR ≦ 5min | 113 | 74 | - | - | - | - | 3 |
| Zhang M et al 2010[40] | Nantong, Jiangsu | Adults and children | 59.25 | NS | Start CPPR > 5min | 341 | 29 | - | - | - | - | 3 |
| Zhang P et al 2010[41] | Shenzhen, Guangdong | Adults and children | 54.55 | NS | All | 297 | 10 | - | - | - | - | 3 |

Continue

|  | Area | Age | Male (%) | B-CPR (%) | Subgroup | Participants (n) | ROSC (n) | Survival to admission (n) | Survival to discharge (n) | One-month survival (n) | Good neurological outcomes (n) | NOS  Score |
| --- | --- | --- | --- | --- | --- | --- | --- | --- | --- | --- | --- | --- |
| Zhang et al 2013[42] | Daqing, Heilongjiang | Adults and children | 68.53 | 0.59 | All | 340 | 10 | - | - | - | - | 4 |
| Zhang et al 2015[43] | Wuxi, Jiangsu | NS | NS | 22.32 | All | 1658 | 73 | - | - | - | - | 4 |
| Zhang et al 2015[43] | Wuxi, Jiangsu | NS | NS | 22.32 | T-CPR | 370 | 19 | - | - | - | - | 4 |
| Zhang et al 2015[43] | Wuxi, Jiangsu | NS | NS | 22.32 | No T-CPR | 1288 | 54 | - | - | - | - | 4 |
| Zhang et al 2018[44] | Taiyuan, Shanxi | Adults | 67.17 | NS | All | 530 | 52 | - | 17 | - | - | 5 |
| Zheng et al 2013[45] | Guangzhou, Guangzhou | Adults | 54 | NS | All | 100 | 19 | - | - | - | - | 4 |
| Zhou et al 2017[46] | Chengdu, Sichuan | Adults | 59.62 | NS | All | 156 | 32 | - | - | - | - | 3 |
| Zhou et al 2017[46] | Chengdu, Sichuan | Adults | 59.62 | NS | Mouth-to-mouth | 52 | 6 | - | - | - | - | 3 |
| Zhou et al 2017[46] | Chengdu, Sichuan | Adults | 61.54 | NS | Trachea cannula | 52 | 9 | - | - | - | - | 3 |
| Zhou et al 2017[46] | Chengdu, Sichuan | Adults | 57.69 | NS | Ballmoon mask | 52 | 17 | - | - | - | - | 3 |
| Zhou et al 2019[47] | Foshan, Guangdong | Adults | 53.17 | 46.03 | All | 126 | 11 | - | 0 | - | - | 5 |
| Zhou et al 2019[47] | Foshan, Guangdong | Adults | 53.44 | 46.03 | T-CPR | 58 | 9 | - | 0 | - | - | 5 |
| Zhou et al 2019[47] | Foshan, GuangdongS | Adults | 52.84 | 46.03 | No T-CPR | 68 | 2 | - | 0 | - | - | 5 |
| Zhou et al 2020[48] | Zhuhai, Guandong | Adults | 69.23 | 1.99 | All | 1612 | 41 | 13 | 3 | 2 | - | 6 |
| Zhu et al 2012[49] | Tangshan, Hebei | NS | 54.63 | 43.52 | All | 108 | - | - | 8 | 5 | - | 5 |
| Zhu et al 2012[49] | Tangshan, Hebei | NS | 54.63 | 43.52 | T-CPR | 47 | - | - | 7 | 5 | - | 5 |
| Zhu et al 2012[49] | Tangshan, Hebei | NS | 54.63 | 43.52 | No T-CPR | 61 | - | - | 1 | 0 | - | 5 |

Note: AED, automated external defibrillator; ROSC, return of spontaneous circulation; NS, not specified; CPR, cardiopulmonary resuscitation; T-CPR, telephone cardiopulmonary resuscitation.

**References**

1. Jiaji Cai HH, Jiongyu Z. Effect of machinical cardiopulmonary resuscitation in patients with prehospital cardiac arrest. Inner Mongolia Medical Journal. 2019;51:326-8.

2. Yingxin Cen SZ, Yanzhang Shu LL. Investigation of out-of-hospital cardiac arrest in Zhengzhou City and the risk factors of prognosis of cardiopulmonary resuscitation: case analysis for 2016-2018. Chinese Emergency Medicine for Critical Illness. 2019;31:439-43.

3. Chunli Chang SL, Li X. Prevention and cure of pre-hospital sudden death: a clinical analysis of 321 cases. Medical Journal of Qilu. 2011;26:240-2.

4. Wenyuan Chen QT, Yingjian Zhang ZW, Shaohui Liu MH, Zhenhe Gao WX. The outcomes of cardiopulmonary resuscitation among 220 patients with cardiopulmonary arrest. Modern Chinese Doctors. 2013;51:137-8.

5. Zhigang Chen MW, Chen Qiu XJ, He B. Characteristics of Pre-hospital treatment of 596 cases of cardiac arrest. Disaster Medicine and Rescue. 2016;5:16-9.

6. Min Chen JH, Cong L. Analysis of influencing factors on the success rate of prehospital cardiopulmonary resuscitation. Medical Frontier. 2019;9:220-1.

7. Yuling Chen PY, Ying Wu JL, Yanni Lei DG, Jiang Liu PH. Trend in survival after out-of-hospital cardiac arrest and its relationship with bystander cardiopulmonary resuscitation: a six-year prospective observational study in Beijing. BMC Cardiovasc Disord. 2021;21:1-13.

8. Jun Dong FL. The influence of 2010 AHA guidelines on the success rate of prehospital cardiopulmonary resuscitation in Shanghai. For all Health. 2013;7:1-3.

9. Pinye Feng JZ, Yang W. Analysis of the status quo and related factors of emergency treatment for patients with prehospital cardiac arrest. Journal of Youjiang Medical Universtity for Nationalities. 2011;33:806-8.

10. Jiansheng Feng BL, Chen Y. Comparisons of Clinical Effectiveness and Survival between Continuous and Interrupted Chest Compressions in Out-of-hospital Cardiac Arrest. Acta Academiae Medicinae Sinicae. 2018;40:473-80.

11. Yingjun Ge MC, Keyu Sun XL, Lijing Jiang YL. The current status analysis of bystander －initiated cardiopulmonary resuscitation for out －of －hospital cardiac arrest. Chinese Journal of Critical Care Medicine. 2015;35:477-80.

12. Jingdong Hu CY, Qun Zhang XW, Wang E. Retrospective analysis of Pre-hospital Cardiopulmonary Resuscitation about 1432 case of sudden deaths. Journal of Anhui Health Vocational and Technical College. 2010;9:27-8.

13. Jingchun Hu XL, Cheng Yang ZW, Wentao Zhang QZ, Wang E. Prehospital cardiopulmonary resuscitation condition difference on the cardiac arrest patient curative effect. Journal of Anhui Health Vocational and Technical College. 2013;12:27-8.

14. Xiaolan Li SZ. Retrospective analysis of 102 cases of Pre-hospital Cardiopulmonary Resuscitation. Sichuan Medical Journal. 2014;35:1219-20.

15. Minghua Li ZX, Jie jiang FL, Ping Xu JY, Jing Ye YL. Analysis of related of ROSC and discharge survival reat in 117 Pre-hospital cardiopulmongary resuscitation cases in Shanghai. Chinese Journal of Critical Care Medicine. 2017;37:603-6.

16. Youqing Li QQ, Xinbing Zhang CJ, Bin Xiong. Analysis of influencing factors of CPR. Journal of Guangdong Medical University. 2018;36:600-2.

17. Liping Li MX, Wu S. Treatment effect of emergency dispatch telephone guidance for cardiopul monary resuscitation on patients with sudden prehospital death. China Medical Herald. 2019;16:139-42.

18. Effect of advanced life support on prehospital emergency cardiac arrest. Cardiovascular Disease Electronic Journal of integrated traditional Chinese and Western Medicine. 2019;7:44-5.

19. Xiaoquan Li XZ. A Retrospective Analysis of Effect of Dopamine combined with Epinephrine on Recovery Rate of Spontaneous Circulation in Pre⁃ hospital Cardiac Arrest Patients. Lingnan Journal of Emergency Medicine. 2020;25:214-6.

20. Haishan Li CW, Hongyuan Zhang FC, Shuang Zuo LX, Hui Chen XW. Evaluation of abdominal compression-decompression combined with chest compression CPR performed by a new device: Is the prognosis improved after this combination CPR technique. Scand J Trauma Resusc Emerg Med. 2022;30:49.

21. Liu Z. Analysis of the important factors influencing the pre-hospital rescue of sudden death patients. China Medical Herald. 2010;7:135-6.

22. Libin Lu SZ. Investigation on the current situation of pre － hospital care for patients with cardiac arrest: epidemiological investigation and analysis of outcomes of patients with out － of － hospital cardiac arrest in Zhengzhou. Henan Medical Research. 2019;28:1356-62.

23. Lingling Lu SD, Yang L. Prehospital Sudden Death Situation Analysis and First Aid Intervention of Zhuhai City. Chinese and Foreign Medical Research. 2013;11:21-2.

24. Xiaoming Rao JX, Zhongmin Huang JL. Application of Autopulse resuscitator in pre hospital seamless cardiopulmonary resuscitation. Journal of Hubei University of Medicine. 2012;31:451-2.

25. Shao F, Li H, Ma S, Li D, Li C. Outcomes of out-of-hospital cardiac arrest in Beijing: a 5-year cross-sectional study. BMJ Open. 2021;11:e041917.

26. Hong Shen TN, Xin Wu MC, Weijun Zhou EC. Clinical effect of two different airway managements on pre-hospital ardiopulmonary resuscitation. Occupational Health and Emergency Rescue. 2021;39:98-102.

27. Geng Su DK, Chen Y. Application and influence of different ventilation methods in patients with prehospital cardiac arrest. Chinese Journal of Modern Drug Application. 2015;9:82-3.

28. Geng Su DK, Chen Y. Influencing factor analysis for the effect of pre -hospital cardio -pul

monary resuscitation. China modern medicine. 2015;22:30-2.

29. Guangming Sun DF, Hongqi Ma RK, Yang W. Emergency treatment analysis of 268 cases of sudden death before hospital. China Practical Medicine. 2013;8:96-7.

30. Hui Sun LX, Chuanyun Qian JX, Xuemei Li YL. Analysis of 2 430 cases of pre-hospital CPR of Kunming City. Medicine and Pharmacy of Yunnan. 2013;34:376-9.

31. Zhuo Wang QZ, Zhang W. Retrospective analysis of 494 cases of cardiac arrest outside hospital. China Journal of Emergency Resuscitation and Disaster Medicine. 2016;11:69-71.

32. Wang Y. Rescue experience of 334 cases of prehospital cardiac arrest. Today Nurse. 2010;12:1-2.

33. Xiaogang Wang DG. The clinical experience of CPＲ in the treatment of 505 cardiac arrest patients Prehospital and the analysis of the influence factor of successful CPＲ. Chinese Journal for Clinicians. 2015;43:39-41.

34. Yu Wang QZ, Guangbo Qu FF, Xiaokang Dai LY, Zhang H. Effects of prehospital management in out-of-hospital cardiac arrest: advanced airway and adrenaline. BMC Health Serv Res. 2022;22.

35. Wu Y. Effect of different cardiopulmonary resuscitation procedures on prehospital emergency cardiac arrest. Proceedings of 2015 Clinical Acute and Critical Care Experience Exchange Summit Forum. 2015:861-861.

36. Bo Xu QH, Hong Zhu JH, Zhou Q. Epidemiological Characteristics and Analysis of Resuscitation Effect for Out-of-hospital Cardiac Arrest in Shenzhen under the Utstein Model. Lingnan Journal of Emergency Medicine. 2020;25:217-20.

37. Haiping Yang LZ, Hongyan Li XW. Epidemiological analysis and emergency treatment of 129 patients with prehospital cardiac arrest. Chinese Journal of General Practice. 2010;13:1324-6.

38. Wei Yuan YW. Effect of different cardiopulmonary resuscitation procedures on prehospital emergency cardiac arrest. Chinese Journal of Gerontology. 2013;33:2635-6.

39. Xiaofan Zhang XH, Yu Zhang YC. Analysis of the current situation of pre-hospital emergency treatment for patients with cardiac arrest in WuXi. Chinese General Practice. 2010;3:2250-1.

40. Ming Zhang HH, Gong X. Analysis on the factors of success and failure of 454 cases of pre hospital cardiopulmonary resuscitation and the intervention strategy. Chinese Journal of General Practice. 2010;13:1322-6.

41. Zhang P. Clinical experience in rescuing 297 cases of sudden death before hospital. Clinical Medicine and Engineering. 2010;17:50-1.

42. Quanxiao Zhang YZ, Hou C. Epidemiological Information Research of Prehospital Sudden Death Characteristics and Clinical Analysis of CPR. Hei Long Jiang Medical Journal. 2013;37:537-9.

43. Xiaofan Zhang JC, Chen K. The current status analysis of medical priority dispatching system in out －of －hospital cardiac arrest event. Chinese Journal of Critical Care Medicine. 2015;35:188-92.

44. Zhang J. Analysis of cardiopulmonary resuscitation effect and influencing factors in patients with out of hospital cardiac arrest. Journal of Clinical Medical Literature. 2018;5:25-8.

45. Zheng J. Observation on the effect of balloon and endotracheal intubation assisted respiration in pre hospital emergency treatment of cardiac arrest. Journal of Hainan Medical. 2013;24:1029-30.

46. Zhou Y. Effect and prognosis of cardiopulmonary resuscitation with different modes in prehospital emergency cardiac arrest. Journal of Practical Medical Techniques. 2017;24:211-2.

47. Jianyi Zhou SL, Zhixin Wu JX, Rong Li JC. Effect of cluster telephone guided cardiopulmonary resuscitation on prognosis of out of hospital cardiac arrest patients. Journal of clinical rational drug use. 2019;12:150-2.

48. Ruiyun Zhou FL, Yang L. Analysis of epidemiological characteristics and treatment of pre-hospital cardiac arrest in Zhuhai City. Shanxi Medicine of Journal. 2020;49:3382-4.

49. Aihua Zhu YD. Clinical effect of on-site cardiopulmonary resuscitation guided by telephone. West Ching Medical Journal. 2012;27:1228-9.
